# Un cas historique d’un nævus fronto-orbito-calvarial

**DOI:** 10.11604/pamj.2023.44.161.30281

**Published:** 2023-04-05

**Authors:** Zahra Sayad, Boulaadas Malik

**Affiliations:** 1 Service de Chirurgie Maxillo-faciale et Stomatologie, Centre Hospitalier Universitaire Ibn Sina, Faculté de Médecine et de Pharmacie, Université Mohammed V Rabat, Rabat, Maroc

**Keywords:** Géant, nævus, scalp, traitement, Giant, nevus, scalp, treatment

## Abstract

We report the case of a 22-year-old female patient, with no particular previous history, who had nevus progressively increasing in volume since birth. Physical examination revealed a huge bluish-grey mass in the left fronto-temporal-parietal region, extending beyond the midline, infiltrating the palpebral region with orbital extension and blindness in the left eye (A). Another isolated nevus was found in the occipital region, which measured 5cm along its longer axis. Craniofacial CT scan with IV contrast showed nevus extension into the left intraorbital region and infiltration of the scalp opposite the nevus, without involvement of the brain parenchyma. A 3-stage surgical procedure was performed, in the first stage two expanders were placed in the healthy occipital and contralateral parietal region. In the second stage, the entire parieto-temporo-occipital component was removed, the frontal component was reduced and the scalp, which had gained enough elasticity thanks to the expanders, was reconstructed using transposition flaps (B). The third stage consisted of resection of the remaining upper fronto-palpebral component and part of the orbital component with repair by total skin graft to cover any loss of substance (C). The result obtained was extraordinary and the patient was so happy to be able to live a normal life.

## Image en médecine

Nous rapportons le cas d´une jeune de 22 ans, sans antécédents particuliers, qui présente depuis la naissance un nævus qui augmentait progressivement de volume. L´examen physique a objectivé une énorme masse bleu-grisâtre de la région fronto-temporo-pariétale gauche, dépassant la ligne médiane, infiltrant la région palpébrale avec une extension orbitaire et une cécité de l´œil gauche (A). On a trouvé aussi la présence d´un autre nævus isolé de la région occipitale faisant 5cm de grand axe. Une tomodensitométrie crânio-faciale injectée a objectivé une extension du nævus en intra-orbitaire gauche ainsi qu´une infiltration du scalp en regard du nævus, sans atteinte du parenchyme cérébral. La prise en charge de cette malade a été chirurgicale étalée sur 3 étapes, en 1^er^ temps on a mis en place deux expandeurs au niveau de la région occipitale et pariétale controlatérale saines. En 2^e^ temps on a réalisé l´exérèse de toute la composante pariéto-temporo-occipitale ainsi qu´une réduction de la composante frontale et la reconstruction par les lambeaux de transposition du cuir chevelu qu´on a gagné suffisamment d´élasticité grâce aux expandeurs (B). Le 3^e^ temps consistait à la résection de la composante restante fronto-palpébrale supérieure et une partie de la composante orbitaire avec réparation par d´une greffe de peau totale pour couvrir l´éventuelle perte de substance (C). Le résultat final est extraordinaire et la patiente est tellement contente de se retrouver capable de mener une vie normale.

**Figure 1:**
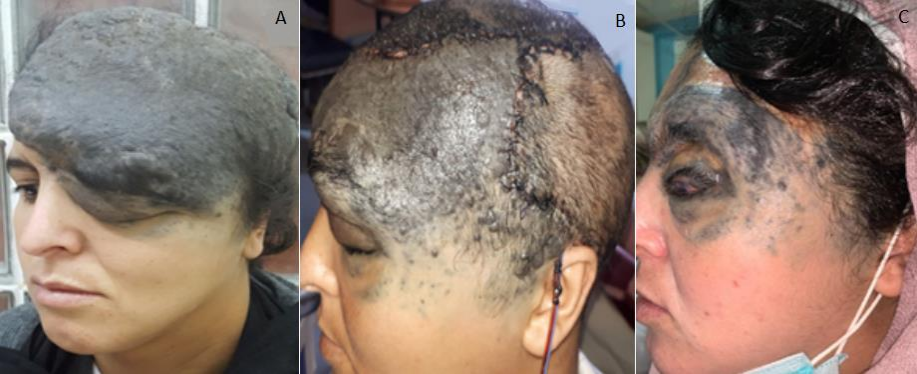
A) photo ¾ montrant un énorme naevus fronto-temporo-pariétal gauche; B) photo de profil post opératoire après 2^e^ temps chirurgical; C) photo de profil quelques semaines après 3^e^ temps chirurgical

